# Transdermal 17β-Estradiol for the Treatment of COVID-19: Protocol of an Early Terminated Phase 2 Randomized Controlled Trial

**DOI:** 10.2196/81541

**Published:** 2026-09-11

**Authors:** Adel Ganaw, Kayleigh Griffiths, Moad Ehfeda, Ashraf Elmalik, Manoj Varghese, Muna Al Maslamani, Prem Chandra, Hugh Montgomery, Melanie Madhani

**Affiliations:** 1 Hamad Medical Corporation, Doha, Qatar; 2 Cardiovascular Department, University of Birmingham, Edgbaston, Selly Oak, Birmingham, B15 2TT, United Kingdom, +44 (0)121 414 3344; 3 Department of Medicine, University College London, London, United Kingdom

**Keywords:** 17β-estradiol, COVID-19, estrogen, immunomodulation, SARS-CoV-2, postmenopausal, safety, feasibility

## Abstract

**Background:**

Early epidemiological studies suggested that pre- and postmenopausal women receiving estrogen therapy were less likely to develop severe disease or die from COVID-19 infection. Potential mechanisms include estrogen-mediated immunomodulation and 17β-estradiol–induced downregulation of angiotensin-converting enzyme type 2 (ACE2), the cellular receptor for SARS-CoV-2.

**Objective:**

This study aimed to evaluate the feasibility, safety, and preliminary efficacy of transdermal 17β-estradiol as an adjunctive treatment for COVID-19 in men and postmenopausal women.

**Methods:**

We designed and conducted a randomized controlled trial comparing 17β-estradiol transdermal gel plus standard care with standard care alone in adults with confirmed COVID-19. Initial ethics and funding approvals were obtained in March 2021. Owing to changes in the epidemiology of COVID-19 in Qatar and revisions to national quarantine policies, protocol amendments were required before recruitment commenced in February 2022. The treatment duration was reduced from 10 to 7 days due to changes in national quarantine guidelines. Recruitment and follow-up were conducted between February 2022 and June 2022.

**Results:**

Recruitment was substantially lower than anticipated because widespread COVID-19 vaccination, declining disease severity, and revised national quarantine policies markedly reduced the number of eligible hospitalized patients. Consequently, the planned sample size was not achieved, and the study was terminated in June 2022. A total of 29 men with mild COVID-19 were enrolled, with 44.8% (n=13) randomized to standard care and 55.2% (n=16) to transdermal 17β-estradiol plus standard care. The intervention was well tolerated, with no adverse safety signals or thromboembolic events reported.

**Conclusions:**

Although the study was underpowered to assess efficacy because recruitment targets were not achieved, it showed that transdermal 17β-estradiol was well tolerated, with no major safety concerns among enrolled participants. The experience also provided important operational lessons for conducting clinical trials during rapidly evolving pandemics. Adequately powered studies are required to determine whether transdermal estrogen has therapeutic potential against COVID-19, other ACE2-mediated coronavirus infections, or potentially other severe viral illnesses.

## Introduction

Since the emergence of SARS-CoV-2 and the resulting COVID-19 pandemic, effective vaccines and therapeutic options, including antivirals and glucocorticoids, have significantly reduced mortality and rates of severe disease [1]. Nonetheless, infection remains common [2], and severe illness continues to pose a serious risk, especially among immunocompromised individuals and those with multiple comorbidities [3]. In this context, low-cost, widely accessible therapeutic alternatives may still offer clinical value. Transdermal estrogen may represent one such candidate.

Epidemiological studies have consistently shown sex-dependent differences in COVID-19 severity and fatality, with male patients more likely to develop severe illness, require hospitalization, and die than premenopausal female patients [4,5]. The mechanisms underlying this sex-specific difference are likely multifactorial and include immunological distinctions between male and female patients [6,7]. Among these factors, estrogens appear to play an important role.

Estrogen concentrations are higher during the reproductive years, and the relative protection observed in female patients appears to diminish after menopause [8,9]. Evidence from observational studies further suggests that this protection may partially be restored by estradiol therapy (odds ratio [OR] 0.33, 95 % CI 0.18-0.62; hazard ratio 0.29, 95% CI 0.11-0.76) [10].

Estrogens, and particularly 17β-estradiol, the most potent form, act through receptors on both innate and adaptive immune cells. Estrogen can modulate immune activity in both proinflammatory and anti-inflammatory directions [11], and female patients tend to show stronger innate and adaptive immune responses to pathogens than male patients [11,12]. Furthermore, nonimmunobiological mechanisms may also contribute to a protective role for estrogen. SARS-CoV-2 enters host cells via angiotensin-converting enzyme type 2 (ACE2) receptors, which are expressed across various tissues including the lung, vasculature, heart, kidney, and brain. ACE2 serves both as a cellular receptor for SARS-CoV-2 and as a key regulator of the renin-angiotensin system [13]. Under physiological conditions, ACE2 converts angiotensin II into the vasodilator angiotensin (1-7), which in turn regulates blood pressure [14]. However, SARS-CoV-2 infection downregulates ACE2 expression, thus increasing angiotensin II activity, which drives inflammation, vascular leakage, vasoconstriction, and pulmonary damage [15].

Preclinical studies further support a protective role for estrogen in COVID-19. Application of 17β-estradiol to Vero E6 monkey kidney cells reduced SARS-CoV-2 viral load [16] and attenuated ACE2 expression in human lung epithelial cells [17]. Mechanistically, estrogen modulates the innate immune system to suppress hyperinflammation and mitigate the cytokine storm by downregulating nuclear factor κ B (NF-κB) signaling [18,19]. Within the respiratory system, estrogen upregulates the Mas receptor, activating an anti-inflammatory and tissue-protective axis [20]. Furthermore, estrogen promotes endothelial nitric oxide synthase (eNOS) activity and subsequent nitric oxide production, which in turn improves microvascular endothelial function—a key issue in severe COVID-19 pathophysiology [21-23].

Despite these promising mechanistic and preclinical findings, clinical evidence to support benefit from estrogen therapy in COVID-19 remained limited [24,25], with no human efficacy data reported. We sought to address this issue and initiated a study to determine whether transdermal 17β-estradiol could reduce COVID-19 disease progression in such patients. However, the widespread COVID-19 vaccination program, declining COVID-19 disease severity, and changes to Qatar’s quarantine policy ultimately limited recruitment and prevented trial completion. Here, we present the study protocol, preliminary findings, and key operational lessons to inform future investigations of estrogen-based therapies for COVID-19 and other diseases caused by coronaviruses that use the ACE2 receptor to gain cellular entry.

## Methods

### Ethical Considerations

A favorable ethical opinion was given by the local ethics committee (MRC-05-099) at Hamad Medical Corporation (HMC), Doha, Qatar. All participants gave informed consent to participate. No financial compensation or other incentives were offered for participation. All participants gave informed consent for publication.

### Choice of Drug and Route of Administration

We chose transdermal estradiol administration because this route bypasses hepatic metabolism, produces more stable circulating estradiol concentrations than oral therapy, and is associated with a lower risk of venous thromboembolism (VTE), an important consideration in patients with COVID-19 [26,27]. A daily dose of 3 mg of 17β-estradiol gel (Besins Healthcare) was chosen because it lies within the clinically established therapeutic dose range and is expected to achieve systemic estradiol concentrations sufficient to activate estrogen-dependent modulatory pathways [28]. Treatment was designed for 10 days to encompass the period during which patients were at greatest risk of clinical deterioration and progression to severe disease, while limiting overall estrogen exposure and maintaining a well-established safety profile.

### Design

This was a multicenter, open-label, phase 2 randomized controlled trial conducted at HMC quarantine facilities (Um Garn) and affiliated hospitals in Qatar. The trial was preregistered on ClinicalTrials.gov (NCT04853069) and approved by the HMC Institutional Review Board (MRC-05-099).

Eligible participants provided written informed consent before any study-specific procedures were undertaken. The informed consent discussion was conducted by trained members of the research team and included an explanation of the study objective, study procedures, the rationale for investigating estrogen treatment, potential risks and anticipated benefits, alternative treatment options, and the participant’s right to decline participation or withdraw from the study at any time without affecting their standard of care. Participants were informed that, although no direct clinical benefit could be guaranteed, their participation could contribute to knowledge regarding potential treatments for COVID-19. Participant information sheets and consent forms were available in English, Arabic, Urdu, Hindi, Malayalam, Tagalog, Bangla, and Nepali to ensure comprehension across the study population.

For participants lacking decision-making capacity at the time of enrollment, assent was obtained from a legal representative or next of kin, with retrospective consent sought from the patient upon recovery.

### Study Setting and COVID-19 Pandemic Response in Qatar

The study was designed in June 2020, a period of high COVID-19 transmission in Qatar, when all individuals testing positive for SARS-CoV-2 were admitted to dedicated isolation quarantine facilities or hospitals (Hazm Mebaireek Hospital, Cuban Hospital, Al-Wakra Hospital, Communicable Disease Center, Messaied Hospital, Ras Laffan Quarantine Center, and an Um Garn quarantine center) regardless of disease severity, providing a large pool of potentially eligible participants. Recruitment commenced in February 2022 following regulatory approval; however, the epidemiological landscape changed substantially during this period. In December 2020, Qatar implemented a rapid national COVID-19 vaccination program, resulting in a marked decline in severe disease and COVID-19–related hospital admissions [29]. In parallel, national quarantine policies evolved from mandatory institutional isolation to home isolation for most individuals with mild disease, substantially reducing the number of hospitalized patients available for screening. Consequently, both the number of eligible participants and the overall severity of illness among hospitalized patients declined considerably during the recruitment period, leading to slower-than-anticipated enrollment and eventual premature termination of the study.

### Study Population

Eligible participants were adults (aged ≥18 years) with confirmed or clinically diagnosed acute COVID-19. Only male adults and postmenopausal women (amenorrhea for >12 months without other causes) were included.

The exclusion criteria were as follows:

- Women taking estrogen supplements or estrogen receptor antagonists
- Abnormal genital bleeding
- History of breast, endometrial, or ovarian cancer
- Untreated endometrial hyperplasia
- Use of lamotrigine
- Thromboembolic disorders (eg, protein C or protein S deficiency or antithrombin III deficiency) or a prior thromboembolic event (including deep vein thrombosis [DVT], thromboembolic stroke, or pulmonary embolism)
- Preexisting liver or renal disease
- Allergy to exogenous estrogens
- Porphyria
- Participation in another interventional clinical trial

### Randomization and Intervention

Participants were randomized 1:1 to receive either standard care or standard care plus 3 mg of 17β-estradiol gel daily, applied to the forearm, upper arm, and shoulder. Randomization was computer-generated using sealed envelopes and stratified by sex and illness severity into 2 strata: stratum 1, mild illness (not requiring hospitalization); and stratum 2, moderate illness (requiring hospitalization with or without oxygen) or severe illness (requiring admission to an intensive care unit [ICU]).

Treatment began within 48 hours of enrollment. The original treatment period was 10 days but was later reduced to 7 days in response to changes in national quarantine policy. Patients discharged from hospitals could complete treatment in quarantine centers.

### Baseline Assessments

Upon recruitment, the following measures were recorded (Table 1):

- SARS-CoV-2 polymerase chain reaction (PCR) results
- Demographics (age, sex, ethnicity, height, and weight)
- Vital signs (temperature, blood pressure, heart rate, respiratory rate, peripheral oxygen saturation [SpO_2_], and fraction of inspired oxygen [FiO_2_])
- Medical history, medications, and allergies
- Date of symptom onset

**Table 1.** Baseline characteristics of the study population in the estrogen and control groups.

Characteristics	Estrogen group (n=16)	Control group (n=13)	Difference (95% CI)	*P* value
Age (years), mean (SD)	36.3 (10.49)	34.7 (8.66)	1.54 (–5.9 to 8.9)	.67^a^
Sex, n (%)				N/A^b^
Female	0 (0)	0 (0)	0 (0)	
Male	16 (100)	13 (100)	0 (0)	
Comorbidities, n (%)				
Hypertension	13 (81.2)	9 (69.2)	12.02 (–19.5 to 43.6)	.67^c^^,^^d^
Diabetes	2 (12.5)	0 (0)	12.5 (–3.7 to 28.7)	.49^c^^,^^d^
Systolic blood pressure (mm Hg), mean (SD)	140.8 (16.81)	134.2 (8.07)	6.59 (–3.9 to 17.1)	.22^a^^,^^d^
Diastolic blood pressure (mm Hg), mean (SD)	92.81 (10.64)	88.08 (9.05)	4.74 (–2.8 to 12.4)	.21^a^^,^^d^
Peripheral oxygen saturation (%), n (%)				.48^c^^,^^d^
100	4 (25)	2 (15.4)	9.6 (–19.3 to 38.5)	
99	6 (37.5)	7 (53.8)	–16.3 (–52.4 to 19.7)	
98	4 (25)	1 (7.7)	17.3 (–8.4 to 43)	
97	2 (12.5)	3 (23.1)	–10.6 (–38.6 to 17.5)	
Hemoglobin (g/L),^e^ mean (SD)	15.75 (1.21)	15.35 (1.26)	0.40 (–0.62 to 1.43)	.42^a^^,^^e^
Platelet count (×10^9^/L),^f^ mean (SD)	241.0 (15.93)	275.6 (17.03)	–34.6 (–83.2 to 13.92)	.15^a^^,^^f^
White blood cell count (×10^9^/L),^g^ mean (SD)	8.7 (0.36)	8.62 (0.62)	0.08 (–1.3 to 1.5)	.90^a^^,^^g^
C-reactive protein (mg/dL)^h^				
Mean (SD)	0.687 (1.00)	0.388 (0.36)	0.30 (0.33 to 0.93)	
Median (IQR)	0.29 (0.19 to 0.46)	0.24 (0.19 to 0.43)	N/A	.78^i^^,h^
D-dimer^j^				
Mean (SD)	106.2 (62.66)	117.4 (54.13)	11.2 (70.6 to 48.2)	
Median (IQR)	97 (55.5 to 148.8)	110 (73.8 to 172.3)	N/A	.56^i^^,j^

a *P* values computed using *t* test.

b N/A: not applicable.

c *P* values computed using chi-square and Fisher exact test.

d Data were available for 16 participants for estrogen and 13 participants in the control group.

e Data were available for 14 participants in the estrogen group and 14 participants in the control group.

f Data were available for 14 participants in the estrogen group and 11 participants in the control group.

g Data were available for 14 participants in the estrogen group and 12 participants in the control group.

h Data were available for 13 participants in the estrogen group and 12 participants in the control group.

i Mann Whitney *U* test.

j Data were available for 10 participants in the estrogen group and 8 participants in the control group.

### Follow-Up and Outcome Measures

Clinical assessments occurred on days 1, 4, 7, 14, and 28. Investigations included the following: chest imaging (X-ray and computed tomography pulmonary angiography [CTPA]); Doppler ultrasound (limbs); blood tests (lymphocyte count, neutrophil-to-lymphocyte ratio, C-reactive protein (CRP), D-dimer, high-sensitivity troponin T [HsTnT], urea, creatinine, and liver function tests [alanine aminotransferase, ALT, and aspartate aminotransferase, AST]); and serum ACE2 levels (days 0 and 14, if available).

### Primary End Point

The primary end point was COVID-19 disease severity, categorized as mild (no hospital admission), moderate (admitted to a medical unit with or without oxygen), or severe (ICU admission or death).

### Secondary End Points

Secondary end points included hospital mortality, hospital length of stay, admission to and length of stay in the ICU or high-dependency unit (HDU), duration of mechanical ventilation, renal replacement therapy, cause-specific mortality, and time to discharge readiness. The Acute Physiology and Chronic Health Evaluation II (APACHE II) score was recorded on admission to the HDU or ICU, if applicable.

### Safety Measures and Monitoring

Although serious side effects of estradiol occur with long-term use (months or years), the trial duration (7-10 days) posed minimal expected risk. However, all participants were monitored daily for adverse events, adverse reactions, and serious adverse events. Potential side effects included breast tenderness (especially in men), fluid retention, hyperglycemia, changes in liver enzyme levels, and rare dermatologic reactions such as chloasma, erythema multiforme, erythema nodosum, and vascular purpura.

Thrombosis risk was carefully assessed using the following measures:

- The transdermal hormonal route carries a low VTE risk [30].
- The Padua prediction score was used for VTE risk stratification.
- Participants at high-risk (score ≥4) received prophylactic anticoagulation.
- Mild cases (quarantined) were assessed for DVT three times a week. All participants were educated about VTE symptoms.

### Sample Size Determination

At the time of trial design, no randomized controlled trial had evaluated transdermal 17β-estradiol for preventing COVID-19 disease progression. Sample size calculation was thus based on the best available published estimates of disease progression among hospitalized patients with COVID-19 [31]. A clinically meaningful relative risk reduction of 34% was prespecified for the intervention, corresponding to a reduction in disease progression (defined below) from 12% to 8%. Assuming a 2-sided significance level of 0.05 and 80% power, 1864 participants were required.

To allow participant withdrawal and loss to follow-up, the recruitment target was increased to 2000 patients. The sample size was calculated using the formula for comparing 2 independent proportions [32]. Interim safety and design review was planned after enrolling 500 and 1000 patients.

### Statistical Analysis

The statistical analysis described below was prespecified in the study protocol. Baseline characteristics were planned to be summarized as mean (SD) or median (IQR) according to the statistical distribution of the data (assumption of normality assessed using the Shapiro-Wilk test) for continuous data, and as frequencies and associated percentages for categorical variables. Planned between-group comparisons included the unpaired *t* test or Mann-Whitney *U* test for continuous variables, depending on data distribution and homogeneity of variance (assessed using the Fisher-Snedecor test), and the chi-square test or Fisher exact test for categorical variables. All statistical analyses were planned to be 2-tailed, with a *P* value of <.05 considered statistically significant. All statistical analyses were planned to be conducted using statistical software packages SPSS (version 29.0; IBM Corp) and Epi Info (Centers for Disease Control and Prevention).  

### Planned Subgroup Analysis

Prespecified subgroup analyses (by sex and BMI), presence or absence of diabetes, ethnicity, time from onset of symptoms, initial reverse transcription–polymerase chain reaction (RT-PCR) cycle threshold (Ct) value, blood markers of disease severity, and requirement for noninvasive or mechanical ventilatory support) were not performed given the limited sample size incurred by premature termination of the trial.

### Trial Modification and Termination

The original study design assumed approximately 200 new COVID-19 cases per day and an enrollment rate of 30%. Although the trial received ethics and funding approval in March 2021, delays in procurement of the investigational product, together with the rapidly evolving COVID-19 epidemiology in Qatar, resulted in recruitment commencing in February 2022. In the intervening period, vaccination, changes in viral subtypes, availability of disease-modifying agents, and changes to national quarantine policies led to a substantial reduction in hospital admissions and earlier discharge of patients. Consequently, the transdermal 17β-estradiol treatment duration was reduced from 10 to 7 days to reflect updated national isolation guidance. Following review by the data safety monitoring board (DSMB), the trial was terminated in June 2022 because continued recruitment was no longer considered feasible.

## Results

**Figure 1.**
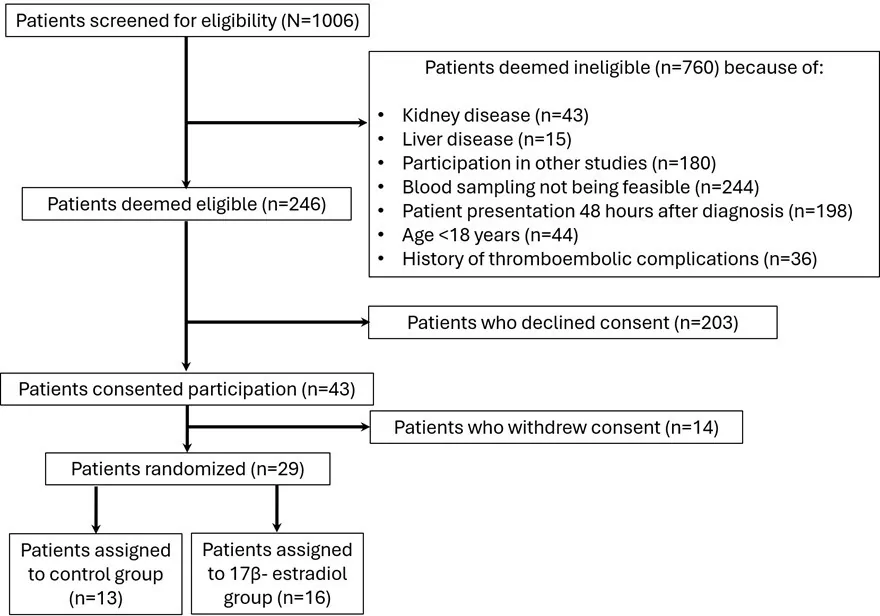
CONSORT (Consolidated Standards of Reporting Trials) flow diagram.

Between February 21, 2022, and June 17, 2022, 1006 patients were screened. Of these, 760 patients were ineligible because of kidney disease (n=43, 5.7%), liver disease (n=15, 1.9%), participation in other clinical trials (n=180, 23.7%), inability to collect timely blood samples (n=244, 32.1%), presentation more than 48 hours after diagnosis (n=198, 26.1%), age <18 years (n=44, 5.8%), or a history of thromboembolic complications (n=36, 4.7%). Additionally, 203 patients declined to provide consent. Of the 43 patients initially eligible, 14 (32.6%) later withdrew consent, leaving 29 (67.4%) participants enrolled in the study (Figure 1).

The 29 enrolled participants were all men of Southeast Asian origin (from Bangladesh, India, Pakistan, Nepal, and Sri Lanka), reflecting the demographic profile of the eligible hospitalized population in Qatar during the recruitment period. Of the 29 enrolled participants, 13 (44.8%) were randomized to standard care and 16 (55.2%) to 17β-estradiol gel plus standard care. Mean age was similar between groups (mean 34.7, SD 8.66 years vs mean 36.3, SD 10.49 years, respectively; *P*=.67), with no statistically significant differences in baseline characteristics (Table 2). The small sample size precluded subgroup analysis. No adverse events, including thromboembolic complications, were reported among participants receiving the 17β-estradiol gel treatment during the 7-day treatment period.

**Table 2.** Summary of the visit structure and schedule of study procedures for the modified 7-day treatment protocol. AG contributed to this table^a^.

Procedures	Screening (Day 1)	Enrollment or baseline: visit 1 (day 1)	Study visit 2 (day 4±1)	Study visit 3 (day 7±1)	Study visit 4 (day 14±1)	Study visit 5 (day 28)
Informed consent	✓					
Demographics	✓					
Medical history	✓					
Randomization	✓					
Study intervention administration		✓	✓	✓		
Concomitant medication review	✓		✓	✓	✓	✓
Physical examination (including height and weight)	✓	✓				
Vital signs	✓	✓	✓	✓		
Height	✓					
Weight	✓					
Severity of disease (mild, moderate, or severe)	✓	✓	✓	✓	✓	✓
Hematology^b^				✓	✓	
Serum chemistry^a^				✓	✓	
Electrocardiogram	✓					
Adverse event review and evaluation	✓		✓	✓	✓	✓
Radiologic or imaging assessment (chest X-ray, computed tomography pulmonary angiography, and ultrasound for the assessment of deep vein thrombosis as indicated)	✓					
Complete case report forms	✓		✓	✓	✓	✓
Reverse transcription–polymerase chain reaction cyclic threshold value	✓				✓	
Acute Physiology and Chronic Health Evaluation II score, if patient was admitted to intensive care unit or high-dependency unit	✓					

a Serum chemistry included blood urea nitrogen, creatinine, liver function tests, high-sensitivity troponin T, and C-reactive protein.

b Hematology included complete blood count, lymphocyte count, neutrophil-to-lymphocyte ratio, and coagulation profile (prothrombin time, partial thromboplastin time, international normalized ratio, fibrinogen, and D-dimer).

## Discussion

Early termination of the trial, due to a rapidly evolving viral, public health, treatment, and vaccination landscape, meant that our study was underpowered to evaluate efficacy. Nevertheless, short-term transdermal 17β-estradiol administration was generally well tolerated in the enrolled participants, and no thromboembolic or other major adverse events were observed during the 28-day follow-up, including VTE or cardiovascular complications. Despite recruitment challenges, the study provides important operational lessons for the design and conduct of clinical trials during rapidly evolving infectious disease outbreaks.

Although preclinical studies have reported beneficial effects of 17β-estradiol in experimental models of COVID-19 [17,22], clinical evidence remains limited. Several trials investigating estrogen therapy for COVID-19 have been registered (NCT04359329, NCT04865029, NCT04539626, and NCT04801836), yet only 2 have reported results to date. Lovre et al [25] reported that a 5-day course of intramuscular estradiol combined with oral progesterone in 10 hospitalized patients with COVID-19 (5 men and 5 women) improved systemic inflammation. In contrast, Foidart et al [24] evaluated another form of estrogen, estetrol, a pregnancy-specific estrogen, in 87 patients with moderate COVID-19 and found that, although estetrol was well tolerated, it did not significantly improve clinical outcomes compared with those in 88 placebo-treated COVID-19 patients. Compared with these studies, our trial evaluated a different estrogen formulation and route of administration. Transdermal 17β-estradiol was selected because it provides continuous systemic delivery while avoiding first-pass hepatic metabolism [33]. Compared with oral estrogen therapy, transdermal administration has less effect on hepatic stimulation of coagulation factors and has been associated with a lower risk of VTE in other clinical settings [27]. However, the present study was not designed or powered to evaluate thromboembolic safety, and therefore no conclusions regarding the comparative risk of VTE can be drawn. Nonetheless, the practical experience gained from this trial provides valuable information to inform the design and conduct of future studies evaluating estrogen-based therapies and other host-directed interventions during infectious disease outbreaks.

Several lessons emerged from this study. First, investigational products and regulatory approvals should be secured as early as possible to minimize delays between protocol development and recruitment. Second, adaptive platform trial designs and methodologies that incorporate prespecified sample size re-estimation or expansion to additional sites may improve resilience to rapidly changing epidemiology. Third, future studies evaluating host-directed therapies such as transdermal 17β-estradiol would benefit from broader recruitment strategies and rapid trial implementation early in an outbreak, when disease incidence is highest, and recruitment targets are more likely to be achieved. Finally, multicenter or multinational recruitment should be considered to reduce dependence on local disease incidence and public health policy.

Of note, a low participation rate was observed among eligible patients. Although 246 patients fulfilled the eligibility criteria, only 43 (17.5%) initially consented to participate, and 14 (32.6%) subsequently withdrew. Selection bias may therefore have occurred, and the study population is not fully representative of the broader population of patients hospitalized with COVID-19.

A further limitation is that reasons for declining participation or withdrawing from the study were not systematically collected. Therefore, it was not possible to determine the factors influencing decisions to decline participation or withdraw from the study. Future clinical trials should prospectively collect reasons for nonparticipation, where feasible, to better understand barriers to recruitment and to inform strategies for improving enrollment.

The study population should also be interpreted within the demographic context of Qatar. All enrolled participants were male expatriate workers from South Asia. This reflects the demographic profile of Qatar, where the population in 2020 was approximately 2.8 million, 72% of whom were male and 83% were aged between 15 and 64 years [29]. Nevertheless, the resulting homogeneous study population limits the external validity of the findings. The observed safety profile cannot be assumed to apply to women, individuals from other ethnic backgrounds, older adults, patients with a higher risk of severe COVID-19, or those with comorbidities. Future multicenter studies recruiting more diverse populations will be important to determine whether the safety and potential efficacy of transdermal 17β-estradiol are consistent across different demographic and clinical groups.

Nevertheless, the biological rationale for investigating 17β-estradiol remains compelling. Sex-specific differences in COVID-19 outcomes have consistently been reported, with men experiencing higher rates of severe disease, intensive care admission, and mortality than women [5]. Estrogen has been shown to modulate innate and adaptive immune responses, reduce inflammatory cytokine production, and regulate ACE2 expression and activity, with the primary receptor used by SARS-CoV-2 for cellular entry [34]. Collectively, these mechanisms suggest that estrogen therapy may attenuate the dysregulated inflammatory responses associated with severe COVID-19. However, the optimal timing of treatment, route of administration, and patient population most likely to benefit remain to be determined. In addition, serial assessments of inflammatory or immunobiological biomarkers are essential to better characterize the biological effects and mechanisms of action of transdermal 17β-estradiol in the context of COVID-19. Finally, larger, adequately powered randomized controlled trials with longer follow-up are warranted to determine both the efficacy and safety of transdermal 17β-estradiol in patients with COVID-19, including its effects on uncommon but clinically important adverse events, such as VTE and cardiovascular complications. Such studies should incorporate mechanistic assessments to further elucidate the immunomodulatory effects of estrogen and identify the patient populations most likely to benefit. If efficacy and safety are confirmed, transdermal 17β-estradiol may have therapeutic potential not only in COVID-19 but also in other ACE2-dependent viral infections.

## Abbreviations

ACE2: angiotensin-converting enzyme type 2
ALT: alanine aminotransferase
APACHE II: Acute Physiology and Chronic Health Evaluation II
AST: aspartate aminotransferase
CRP: C-reactive protein
Ct: cycle threshold
CTPA: computed tomography pulmonary angiography
DSMB: Data Safety Monitoring Board
DVT: deep vein thrombosis
eNOS: endothelial nitric oxide synthase
FiO_2_: fraction of inspired oxygen
HDU: high-dependency unit
HMC: Hamad Medical Corporation
HsTnT: high-sensitivity troponin T
ICU: intensive care unit
NF-κB: nuclear factor κ B
OR: odds ratio
PCR: polymerase chain reaction
RT-PCR: reverse transcription–polymerase chain reaction
SpO_2_: peripheral oxygen saturation
VTE: venous thromboembolism
